# Asymptomatic saccular portal vein aneurysm: a case report and review of the literature

**DOI:** 10.1007/s40477-022-00659-2

**Published:** 2022-02-03

**Authors:** Rosanna Villani, Pierluigi Lupo, Anna Grazia Angeletti, Antonia Federica Sacco, Luca Macarini, Gaetano Serviddio

**Affiliations:** 1 University Centre for Liver Disease Research and Treatment, Liver Unit, Department of Medical and Surgical Sciences, University of Foggia, Foggia, Italy; 2 Department of Radiology, University of Foggia, Foggia, Italy

**Keywords:** Ultrasound, Portal vein, Aneurysm

## Abstract

Portal vein aneurysms are rare abnormal dilations of the portal vein and represent less than 3% of all visceral aneurysms. They may be congenital or acquired, symptomatic or asymptomatic, complicated or uncomplicated. Portal vein aneurysms may be fusiform or saccular and this last one has a low prevalence. Due to the small number of cases reported in the medical literature and the lack of specific guidelines, the management and treatment of this condition is still undefined. In this review, we report a case of saccular portal vein aneurysm in a 73-year old man with liver cirrhosis and discuss all cases of portal vein aneurysms reported in literature.

## Introduction

Portal vein aneurysm is the abnormal dilation of the portal vein and is defined as a portal vein diameter exceeding the 19 mm in cirrhotic patients and 15 mm in normal livers [1].

It was firstly described in 1956 by Barzilai and Kleckner, and since then, only less than 200 cases have been reported [1].

It represents less than 3% of all visceral aneurysms and etiology is still unknown, even if two forms are generally considered: congenital and acquired [2].

The main cause of the acquired form is liver cirrhosis complicated by portal hypertension [3]. In these patients, hyperdynamic circulation or portal vein invasion by malignancy is involved in the pathogenesis.

Portal vein aneurysms may be fusiform or saccular; fusiform aneurysms are more common than saccular aneurysms, however these last ones are more likely to have complications [4].

We report a case of saccular portal vein aneurysm in a 73-year old male with alcohol-related cirrhosis. Portal vein aneurysm was acquired, uncomplicated and, moreover, the patient was asymptomatic.

We also performed a systematic literature search in PubMed and Scopus databases and discussed all case reports dealing with portal vein aneurysms.

## Case description

**Fig. 1:**
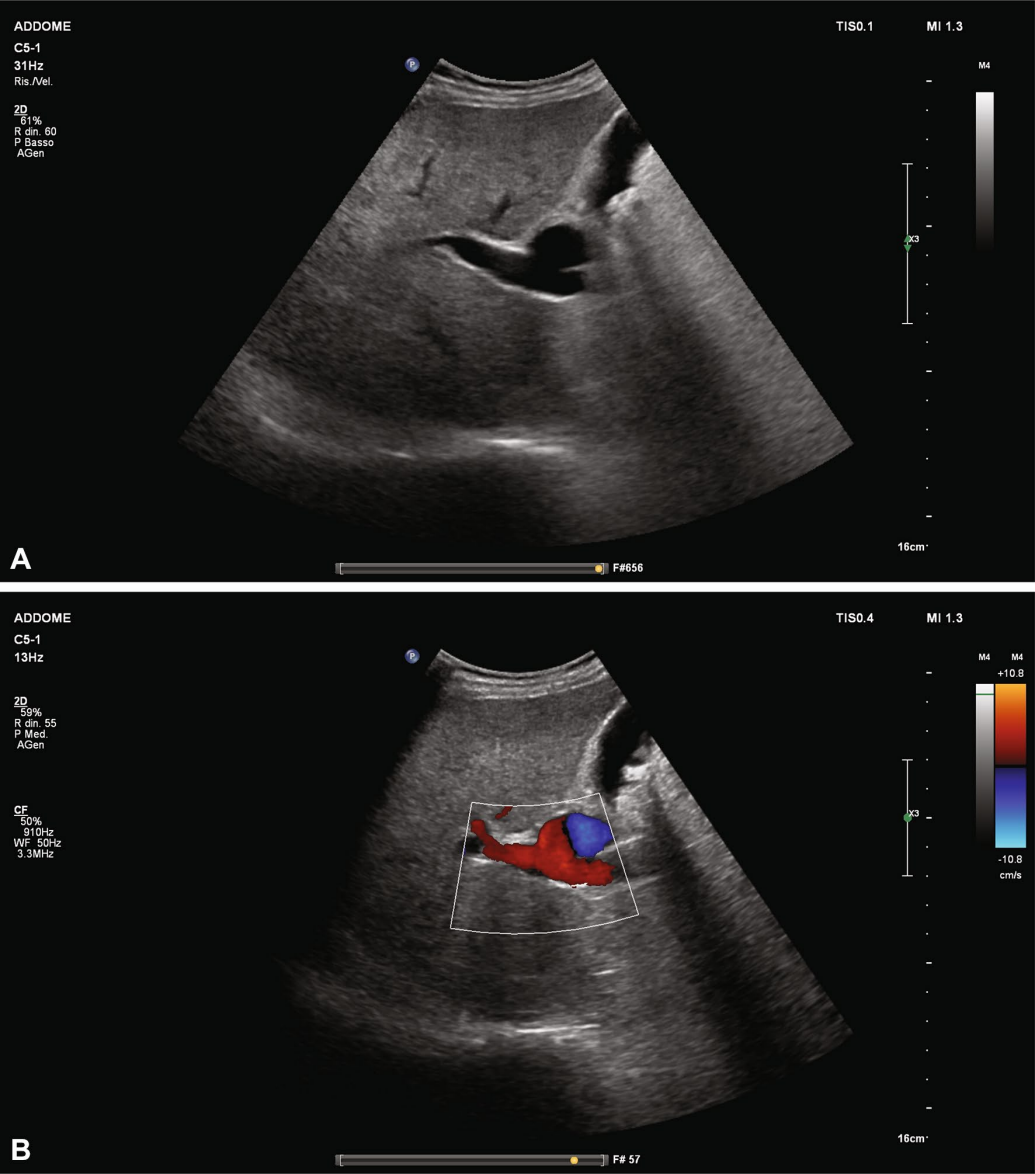
B-mode ultrasound (**A**) and Color Doppler imaging (**B**) show the saccular aneurysm of the distal portal vein. **B** show the “Yin-Yan” sign resulting from the bidirectional flow to and from the saccular aneurysm

**Fig. 2:**
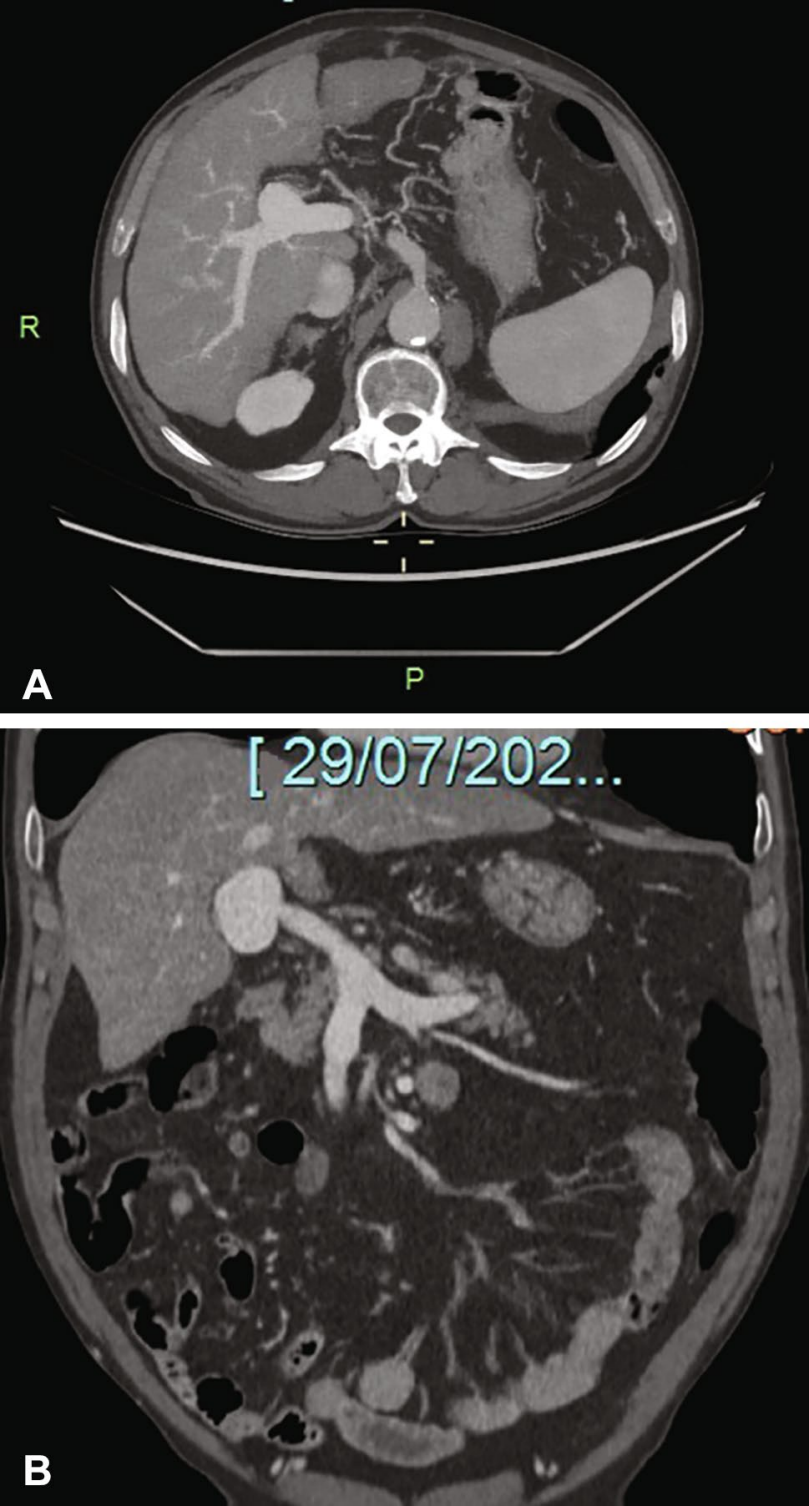
Two-dimensional CT imaging. Transverse (**A**) and coronal (**B**) sections are shown

**Fig. 3:**
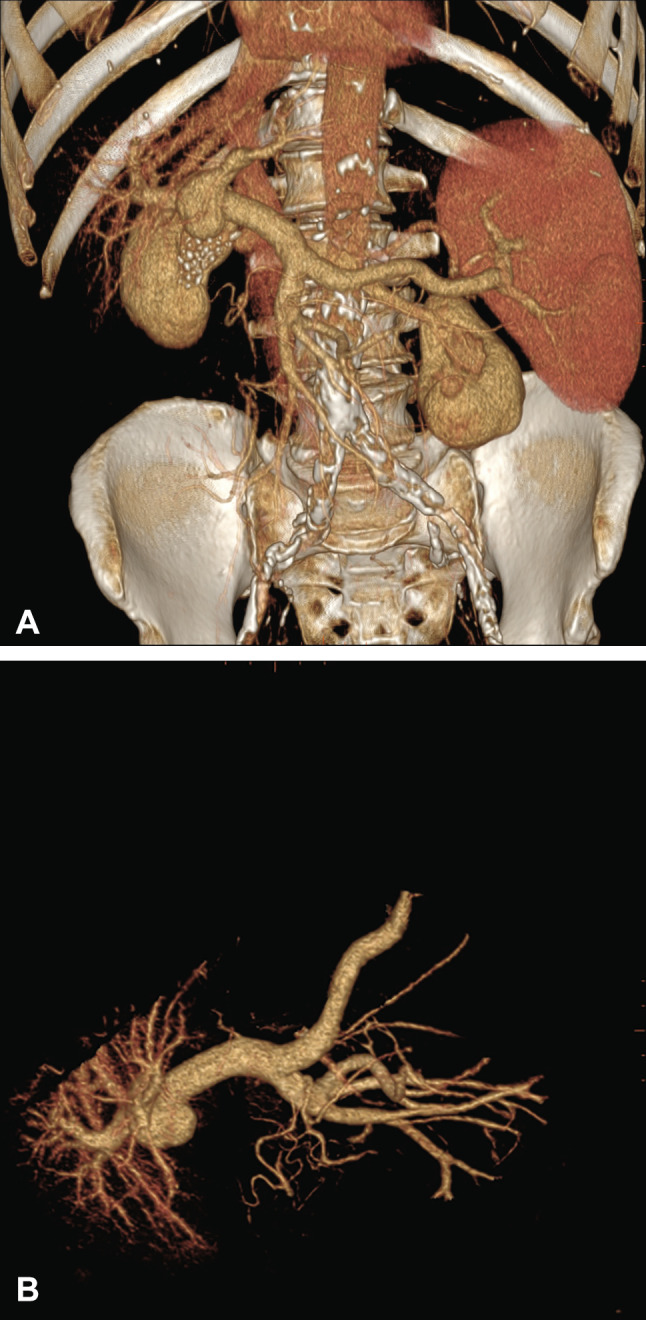
Abdominal CT angiography with 3D reconstructions show the portal vein system and the saccular aneurysm of the distal segment of the portal trunk (**A** anterior view; **B** lateral view)

A 73-year old male patient presented to the outpatient clinic of our Liver Unit. Patient’s medical history included excessive alcohol intake (at least 10 drinks in a day for more than 10 years), moderate splenomegaly and thrombocytopenia (112.000/µL). Abdominal ultrasonographic examination revealed a hepatic coarse echo pattern, narrowed hepatic veins and increased portal vein diameter (14 mm). An hypoechoic area with a maximal diameter of 40 mm was detected next to the main trunk of the portal vein. A definite communication between this lesion and the main branch of the portal vein was observed. Color Doppler evaluation showed bidirectional, swirling, non-pulsatile and monophasic flow (“Korean flag” or “Yin-Yang” sign) within the hypoechoic area (Fig. 1). No signs of portal vein thrombosis were observed, whereas splenomegaly and mild ascites were also found. 2D and 3-D abdominal CT imaging confirmed the diagnosis (Figs. 2, 3).

## Discussion

Portal venous aneurysms represent approximately 3% of all venous aneurysms with a reported overall prevalence of 0.06% [2, 5]. The incidence has been increasing over time thanks to the use of modern imaging techniques in clinical practice.

Portal vein aneurysms are generally asymptomatic and diagnosed as incidental findings during ultrasound examinations. They can be congenital or acquired and various complications such as biliary tract compression, portal vein thrombosis, and rupture may occur over time [1].

Congenital aneurysms may develop after incomplete regression of vitellin vein because the development of portal vein is linked to the involution of the interconnections between the right and left vitelline veins. The incomplete involution of the distal part of the vitellin vein, followed by the formation of a diverticulum of the portal wall or the presence of a defect in the portal wall [6, 7] may be involved in the physiopathology of portal vein aneurysms. These theories are supported by the observation of congenital aneurysms in children [1].

On the other hand, chromosomal disorders seem not to be associated with portal vein aneurysm development. For this purpose, only one case by Sari et al. described a portal vein aneurysm in a 19-year old man with Klinefelter syndrome and abdominal pain, nausea and vomiting [8].

Acquired aneurysms are generally secondary to portal hypertension in patients with weakening of the venous wall due to high splanchnic flow and hyperdynamic circulation, severe pancreatitis, trauma or invasion of the portal vein wall by malignancy [9–12].

A potential role of cholecystitis and choledocholithiasis in the portal vein aneurysm development has been suggested by some authors [13], but these data have not been confirmed.

The acquired forms may affect the main portal trunk, portal bifurcation and intrahepatic portal branches and are generally asymptomatic (about 30%) or paucisymptomatic in 50% of cases and associated with mild and nonspecific abdominal pain [3, 14].

Literature data have shown that only less than 10% of patients present with gastrointestinal bleeding, jaundice due to biliary tract compression, duodenal compression or inferior vena cava obstruction, whereas about 20% may be complicated by thrombosis [1].

Only one case reported by Khairallah et al. described the presence of wall calcifications within the intima and media of portal vein aneurysm due to mechanical stress [15]. In patients with portal hypertension, compensatory intimal thickening and medial hypertrophy may be observed. The medial hypertrophy may be associated with the deposition of fibrous tissue that weakens the wall and, rarely, calcifications.

Intrahepatic portal vein aneurysms are generally smaller than extrahepatic aneurysms and no correlations have been demonstrated with age or sex [16].

In 2015 Laurenzi et al. reported, in their overview of the international literature, only 96 case reports including 190 patients with portal vein thrombosis [1]. Liver cirrhosis was found in 26% and portal hypertension in 32% of patients. Concerning the aneurysm localization, about 80% involved the main portal trunk, portal bifurcation or intrahepatic branches, whereas 20% affected the spleno-mesenteric confluence.

In the medical literature, only a few cases reported spontaneous rupture or regression of the portal vein aneurysms. Priadko et al. recently described 3 cases of asymptomatic portal vein aneurysms with no progression in dimension during a long-term follow-up [3].

Treatment options, conservative management or surgery, should be considered case by case.

Currently, in asymptomatic patients with small portal vein aneurysms, conservative management with regular ultrasound follow-up (every 6 months) is acceptable. The management of patients with large but asymptomatic aneurysms is questionable because a surgical approach should be considered taking into account the coexistence of portal hypertension and, if any, other liver-related clinical issues such as decompensation or kidney failure [17].

Surgical treatment is the recommended strategy in cases of symptomatic patients without portal hypertension showing abdominal pain, biliary tract compression, enlarging aneurysms with risk of spontaneous rupture or in case of rupture [18].

In these patients, aneurysmorrhaphy or aneurysmectomy, according to the type of aneurysm (saccular or fusiform), are the treatments of choice. In patients without portal hypertension, these procedures may be considered a definitive treatment because portal vein aneurysm is generally congenital and no liver disease or portal hypertension coexist [1].

In patients with liver cirrhosis complicated by portal hypertension and criteria for surgical management, shunting and liver transplantation are considered as treatment options due to the high risk of surgical mortality. In this case, the aim is to decompress the portal system to prevent the dilation of the aneurysm [1, 17].

Recently, some authors have proposed the use of transjugular porto-systemic shunt (TIPS) or embolization as new treatment approaches.

Ding et al*.* described a case of 70-mm aneurysmal dilation of the distal portal vein involving the left and right branches in a 48-year old man with hepatitis B-related cirrhosis. The patient had undergone a splenectomy because of upper gastrointestinal hemorrhage and, after portal aneurysm diagnosis, he was treated with a TIPS to prevent its progressive dilation. At the 6-month follow-up the aneurysm was decreased and, after 2 years, disappeared [19].

Shukla et al*.* reported the case of a rapidly growing saccular intrahepatic portal vein aneurysm (3 cm) in a 55-year-old man. Percutaneous embolization successfully occluded the aneurysm and prevented further growth and complication [20].

Finally, Tsuo et al. observed a saccular aneurysm at the extrahepatic portal vein main trunk (3 cm) in a 65-year-old woman with Budd–Chiari syndrome and abdominal pain. TIPS was created with a significant decrease in the aneurysm size (1.9 cm after 1 year) [21].

## Conclusion

Portal vein aneurysms are rarely observed in clinical practice, therefore, no definite data are available on natural history, risk of complications and treatment. In all cases reported in literature, abdominal ultrasound played a pivotal role being the first diagnostic approach for the identification, evaluation of size and complications, and long-term follow-up. The use of Color Doppler US with the typical “Korean flag” or “Yin-Yang” flow pattern may provide crucial information and help the identification of portal vein aneurysms and thrombosis. Undoubtedly, ultrasonography and Color Doppler US are excellent tolls for the diagnosis and follow-up of portal vein aneurysms.
